# The Knee Clinical Assessment Study – CAS(K). A prospective study of knee pain and knee osteoarthritis in the general population: baseline recruitment and retention at 18 months

**DOI:** 10.1186/1471-2474-7-30

**Published:** 2006-03-16

**Authors:** George Peat, Elaine Thomas, June Handy, Laurence Wood, Krysia Dziedzic, Helen Myers, Ross Wilkie, Rachel Duncan, Elaine Hay, Jonathan Hill, Rosie Lacey, Peter Croft

**Affiliations:** 1 Primary Care Sciences Research Centre, Keele University, Keele, North Staffordshire, ST5 5BG, UK; 2 Staffordshire Rheumatology Centre, Haywood Hospital, High Lane, Stoke-on-Trent, North Staffordshire, ST6 7AG, UK

## Abstract

**Background:**

Selective non-participation at baseline (due to non-response and non-consent) and loss to follow-up are important concerns for longitudinal observational research. We investigated these matters in the context of baseline recruitment and retention at 18 months of participants for a prospective observational cohort study of knee pain and knee osteoarthritis in the general population.

**Methods:**

Participants were recruited to the Knee Clinical Assessment Study – CAS(K) – by a multi-stage process involving response to two postal questionnaires, consent to further contact and medical record review (optional), and attendance at a research clinic. Follow-up at 18-months was by postal questionnaire. The characteristics of responders/consenters were described for each stage in the recruitment process to identify patterns of selective non-participation and loss to follow-up. The external validity of findings from the clinic attenders was tested by comparing the distribution of WOMAC scores and the association between physical function and obesity with the same parameters measured directly in the target population as whole.

**Results:**

3106 adults aged 50 years and over reporting knee pain in the previous 12 months were identified from the first baseline questionnaire. Of these, 819 consented to further contact, responded to the second questionnaire, and attended the research clinics. 776 were successfully followed up at 18 months. There was evidence of selective non-participation during recruitment (aged 80 years and over, lower socioeconomic group, currently in employment, experiencing anxiety or depression, brief episode of knee pain within the previous year). This did not cause significant bias in either the distribution of WOMAC scores or the association between physical function and obesity.

**Conclusion:**

Despite recruiting a minority of the target population to the research clinics and some evidence of selective non-participation, this appears not to have resulted in significant bias of cross-sectional estimates. The main effect of non-participation in the current cohort is likely to be a loss of precision in stratum-specific estimates e.g. in those aged 80 years and over. The subgroup of individuals who attended the research clinics and who make up the CAS(K) cohort can be used to accurately estimate parameters in the reference population as a whole. The potential for selection bias, however, remains an important consideration in each subsequent analysis.

## Background

Longitudinal observational research provides critical information on the course, causes, and outcomes of rheumatological disorders [1,2]. In the field of knee osteoarthritis, a state-of-the-science evaluation identified 15 existing population-based prospective cohort studies from the United States and Europe [3]. In addition to examining multiple hypotheses that are specified a priori, these studies also constitute a resource for subsequent nested case-control and case-cohort analyses. When questions are raised about the value of longitudinal observational research, they are more often addressed at its quality rather than its value in principle [4].

Selective non-participation and loss to follow-up are important aspects of the quality of longitudinal research conducted in the general population. The choice of general population setting (as opposed to patient cohorts in clinical settings) coupled with a sampling approach that aims to derive a representative sample of a defined reference population are often justified on the grounds of external validity [5]. This applies to study findings concerning both absolute measures of frequency (e.g. the proportion experiencing a particular outcome at a point in time or within a given period of observation) and measures of association (e.g. the relationship between a specific exposure or risk indicator and a particular outcome). Non-participation at baseline (referred to here as the combined effect of non-response and non-consent) and loss to follow-up (attrition) are potential threats to the external validity of study findings. Biasing effects of non-response and attrition will tend to be higher as the proportion of non-respondents and losses to follow-up from the eligible population increases. The extent to which non-responders and those lost to follow-up differ from the population mean on the parameter of interest will also influence the degree of bias [6]. By reducing the sample size there is also a consequent loss of precision in parameter estimates. Given the importance of selective non-participation and loss to follow-up it is understandable that these are included as criteria for appraising the "quality" of observational cohort studies [7-9].

The need to carefully assess these matters in studies of rheumatological conditions has long been recognised. In one of the earliest studies of arthritis, the Pittsburgh Arthritis Study, only 60% of those invited to attend a clinical assessment were successfully examined, prompting efforts to evaluate factors associated with non-participation [10,11]. Although differences between participants and non-participants were discussed, it was recognised that participation may be quite specific to the geographical location, target population, and topic of research. Their main recommendation was that an investigation of non-participation should be included in the plans for every study in which clinical examinations are performed. Unfortunately the quality of many more recent longitudinal studies of hip and knee osteoarthritis has been found to be low [12]. Failing to sufficiently investigate or report non-participation and loss to follow-up may contribute to this.

In this article we describe the result of recruitment and retention at 18 months of participants in a prospective observational cohort study of knee pain and knee osteoarthritis in the general population. This study involved multiple stages of data collection at baseline, providing serial opportunities for self-selection out of the study, but also providing data for tracking differences between respondents and non-respondents. We pay particular attention to the occurrence of selective non-participation and loss to follow-up, and their possible effect on the external validity of absolute measures of frequency and of measures of association within the cohort.

## Methods

### Design

The Clinical Assessment Study (Knee) – CAS(K) – is a population-based prospective observational cohort study in four phases of adults aged 50 years and over, registered with one of three general practices (irrespective of their actual consultation patterns). Ethical approval for the study was obtained from the North Staffordshire Local Research Ethics Committee (Project Reference Numbers: 1430, 03/94). Full details of the study design and methods have been presented elsewhere [13,14]. Briefly, the four phases were:

#### Phase 1: Baseline two-stage mailed survey

A Health Survey questionnaire was mailed to all eligible patients that included measures of socio-demographic characteristics, general health status, psychological and lifestyle variables, and recent pain. Respondents who provided written consent to further contact and who reported knee pain in the past 12 months were sent a Regional Pains Survey questionnaire which collected more detailed data on their reported knee pain including position, laterality, duration, and pain, stiffness and functional limitation using the Western Ontario and McMaster Universities OA index (WOMAC LK 3.0) [15].

#### Phase 2: Baseline clinical assessment study of the knee (CAS(K))

Participants with knee pain completing both of the mailed surveys were sent a letter inviting them to attend a research clinic that included a standardised assessment using digital photographs of the lower limbs and hands, clinical interview and examination of the knees and hands, plain radiographs of both knees and both hands and a brief self-complete questionnaire.

#### Phase 3: 18-month prospective review of general practice medical records

All participants in Phase 1 who gave permission for their GP records to be accessed had their computerised medical records tagged by a member of the Centre's Health Informatics Specialist team. All consultations in the 18-months periods both before and after the baseline clinical assessment were identified. The three practices participating in this research are fully computerised and undergo annual audits completed by the Health Informatics team to assess the quality and completeness of the data entry at the practices.

#### Phase 4: Follow-up mailed survey at 18 months

A follow-up survey was mailed to all Phase 2 participants approximately 18 months after their baseline clinical assessment. Prior to mailing out this follow-up questionnaire, a member of the Health Informatics team accessed the participating general practice registers in order to gain recent contact details for the participants. Those who were found to have left the practice were traced through the NHS tracing service and their new general practitioners were asked for permission to include them in the follow-up. In addition to the standard mailing process used for the baseline questionnaires (three waves: initial mailing, followed by a postcard reminder to non-respondents, and then by a repeat questionnaire to remaining non-respondents), two further contact stages of minimal data collection (MDC) were used for those who still did not respond, first by post and then by telephone call, were used to gain just the primary outcome data (WOMAC scores).

### Statistical analysis

The target population for CAS(K) was adults aged 50 years or over, registered with one of the three participating practices, and reporting knee pain in the previous 12 months. To determine the extent of selective non-participation and loss to follow-up we compared the characteristics of respondents to the population from which they were drawn at each selection point of the recruitment and follow-up. At the initial recruitment point, which was response to the mailed Health Survey, this comparison was based simply on age, gender, and practice distribution. At each subsequent selection point more information became available on which to make these comparisons, allowing selective non-response and non-consent to be evaluated in relation to socioeconomic characteristics, general health, beliefs about joint pain and osteoarthritis, knee pain characteristics, and radiographic disease. Given the number of possible comparisons, this was done in first instance by simple 'eye-balling'. We then summarised and quantified the main selection effects by comparing characteristics in the observed target population (respondents to the Health Survey reporting knee pain within the previous 12 months) with clinic attenders using logistic regression.

The representativeness of the CAS(K) cohort in terms of chronicity and severity of knee pain at baseline was an important consideration. However, the WOMAC was gathered in the second postal questionnaire (Regional Pains Survey). Some selective non-participation may already have occurred prior to this. In addition, therefore, we compared the distributions of these variables in the CAS(K) cohort with the distributions reported in a single-stage postal survey with high response conducted at three separate practices in North Staffordshire in the same age group and using the same case definition and measures of chronicity and severity of knee pain [16,17].

To investigate the effect of selective non-participation on measures of association, we chose to examine the relationship between body mass index (based on self-reported height and weight) and physical function (SF-36 Physical Function scale [18]). A positive association between these two has been reported in previous population studies of the general population and in those with knee pain [17,19]. Using logistic regression, we compared the strength and direction of this association in the observed target population and in CAS(K) clinical attenders at baseline, before and after adjusting for age and gender. Furthermore, we stratified both groups on the basis of features related to selective non-participation and compared the strength and direction of the association between BMI and function within each stratum.

## Results

### Cohort recruitment

#### Phase 1: Baseline two-stage mailed survey

Health Survey questionnaires were posted to all adults aged 50 years and over at the three practices (n = 8984) over the time period 1 July 2002 to 13 May 2003. During the three mailing waves of the questionnaires, 221 exclusions were made to the database (98 deaths or departures from the practice, 104 questionnaires were returned as addressee unknown, and 19 people had comprehension or memory problems), leaving an eligible study population of 8763 adults. 6108 completed questionnaires were received from the eligible 8763, giving an adjusted response of 69.7%. The non-responders were made up of 223 people who declined to participate, 83 people who stated ill health as the reason for their not completing the questionnaire, and 2349 people for whom no response was received. 99.5% of the responding population reported their ethnicity as white.

**Figure 1:**
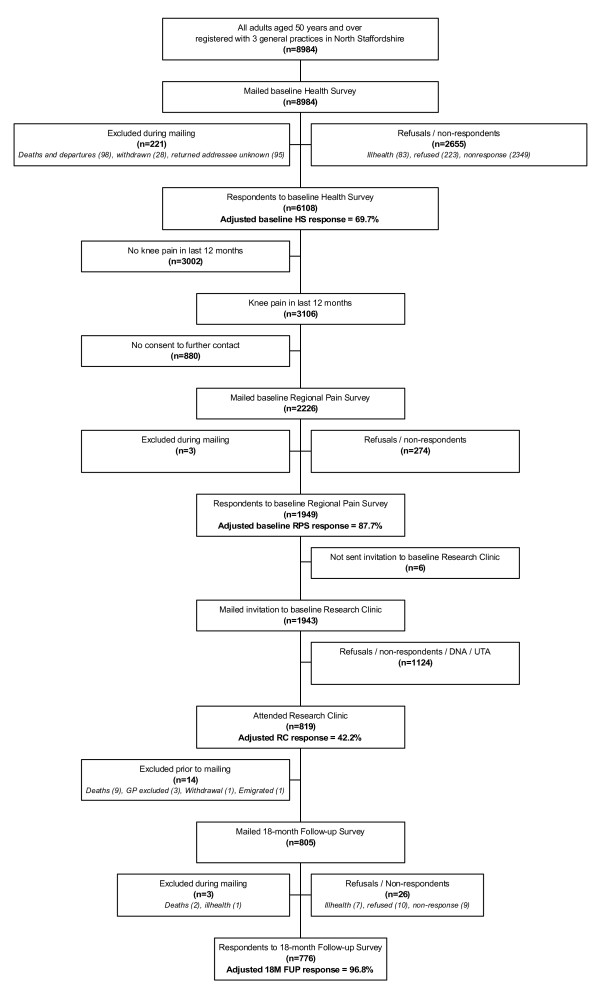
Flow diagram showing recruitment of CAS(K) participants.

Of these 6108 responders, 3106 (50.9%) reported that they had experienced knee pain in the past 12 months (observed target population), of whom 2226 (71.2%) gave written permission to be further contacted and were mailed a Regional Pain Survey questionnaire. During the three mailing waves of this second questionnaire, three exclusions were made to the database (three deaths or departures from the practice) leaving an eligible study population of 2223 adults. 1949 completed questionnaires were received from the eligible 2223, giving an adjusted response of 87.7%. The non-responders were made up of 39 people who declined to participate, 6 people who stated ill health as the reason for not completing the questionnaire, and 229 people from whom no response was received (Figure 1).

#### Phase 2: Baseline clinical assessment study of the knee (CAS(K))

Of the 1949 participants who completed both surveys in Phase 1, 1943 were sent a letter of invitation to the clinical assessment study and 819 participants (42.2%) attended an appointment. Plain radiographs were completed on 790 participants (Figure 1).

#### Phase 3: 18-month prospective review of general practice medical records

Of the 3106 responders to the Health Questionnaire survey 2423 (78.0%) gave permission on the Health Questionnaire survey to access their medical records. This figure was higher amongst the participants attending the clinical assessment at 779 (95.1%).

### Cohort retention

#### Phase 4: Follow-up mailed survey at 18 months

Of the 819 participants that attended the research clinic, 14 exclusions were made to the database prior to mailing the 18-month follow-up questionnaire (9 deaths, three general practitioner exclusions, one moved abroad, one refusal), leaving an eligible study population of 805 adults. During the three mailing waves of the questionnaire, three exclusions were made to the database (two deaths and one due to cognitive impairment), leaving an eligible study population of 802 adults. 776 completed questionnaires were received from the eligible 802 (760 – full questionnaire, 16 – MDC), giving an adjusted response of 96.8%. The non-responders were made up of 9 people who declined to participate, 7 people who stated ill health as the reason for not having completed the questionnaire, one person who was away on long-term holiday and 9 people from whom no response was received (Figure 1). Included in this response were 11 baseline participants who had moved practice during the follow-up period. All were successfully found by the NHS tracing service. One was a GP exclusion, two were non-responders and 8 completed the full questionnaire.

### Selective non-participation and loss to follow-up

#### Practice participation

The representation of the three practices participating in the study was similar at each of the selection points across the 18-month study period (Table 1).

#### Age and gender

Basic demographic information was available for all the eligible population at baseline from the practice register (Table 1). Those participating in the Health Survey questionnaire, who also reported knee pain in the past year, had a similar age distribution to that in the baseline eligible population. However, those who additionally gave permission for further contact were less likely to be aged over 80 years. This age group were also less likely to attend the clinical assessment. The gender distribution of the samples at the various selection points was similar to that seen in the baseline eligible population.

#### Demographic, general health, psychological and lifestyle characteristics

This information was collected on the Health Survey questionnaire and so was only available for the 6108 respondents to this questionnaire (Table 2). Amongst respondents with knee pain, those giving permission for further contact were more likely to be married or co-habiting. In those attending the clinical assessment and completing the 18-month follow-up questionnaire, the percentage of married/co-habiting participants increased again. The proportion of subjects who had attended higher education increased across each of the selection points. Health Survey respondents who were in employment were more likely to consent to further contact but less likely to attend the research clinics. Those in higher managerial jobs were over-represented when compared to the responders to the Health Survey questionnaire. Individuals who were depressed or anxious according to the Hospital Anxiety and Depression Scale [20] were less likely to attend the research clinics; however, this was not reflected in the SF-12 mental component scores which remained stable across all selection points. Participants' views on the seriousness of osteoarthritis and the impact that doctors can have on joint pain were very similar across the respondents at the various selection points.

#### Knee pain characteristics

Detailed information regarding knee pain characteristics were collected at two selection points: baseline Regional Pains Survey questionnaire, and baseline research clinic attendance (Table 3).

Participants with knee pain of less than seven days' duration were under-represented in those attending the baseline clinical assessment study compared to the levels in those responding to the Regional Pains Survey questionnaire. However, scores on the pain, stiffness and physical functioning sub-scales of the WOMAC were similar at all three selection points and almost identical to normative data for the whole population of knee sufferers aged 50 years and over and for each age and gender stratum (Table 4).

Table 5 summarises the main selection effects comparing the characteristics of clinic attenders (n = 819) to those in the observed target population who did not attend the research clinic (n = 2287). Female gender, age 80 years and over, not being married/cohabiting, lower educational attainment, manual occupations, and possible or probable anxiety or depression were associated with non-participation.

#### Association between self-reported BMI and physical function

A positive association between BMI and physical function was observed in the survey respondents reporting knee pain (Table 6). This was stronger after adjusting for age and gender. The same pattern was observed in the sub-group attending clinic, although the association here was marginally stronger (both crude and adjusted odds ratios). We stratified both groups separately by age (50–59, 60–69, 70–79, 80+ years), occupation (manual, non-manual), anxiety (none/possible, probable), and depression (none, possible/probable). Within each stratum, the association between BMI and function tended to be stronger in the CAS(K) clinic attenders than in the observed target population (although there were insufficient numbers in the 80+ and possible/probable depression groups for meaningful analysis; data not shown). The slightly stronger association between BMI and function observed in the CAS(K) clinic attenders as a whole, therefore, is not simply caused by the non-participation of individuals from a particular stratum in whom that association is weak.

## Discussion

In the current study, 819 participants attended the clinical assessment from 1949 invited (42%). However, this is from a potentially eligible population of 3106 with knee pain in the target population (giving a crude response of 26%). Assuming the prevalence of knee pain in the non-responders was the same as that observed in the responders to the Health Survey (50.9%), this would lead to a clinical assessment participation rate of 18.4% from the total surveyed population (n = 8763). In such circumstances there is clear potential for poor representativeness of the subgroup attending the research clinic. The inclusion of multiple stages of data collection at baseline provides serial opportunities for self-selection out of the study but does also provide data for tracking differences between respondents and non-respondents. Taking Health Survey respondents with knee pain as the target population (n = 3106), the main selection effects that were apparent were selective non-participation of persons aged 80 years and over, females, not married/cohabiting, those with lower educational attainment or from lower socioeconomic groups (less likely to consent to further contact and to attend research clinic), those in employment, those experiencing anxiety or depression, or those reporting only a brief episode of knee pain within the previous year (less likely to attend research clinic). Given this pattern, it seems unlikely that any single form of selective non-participation is operating. It is more probable that there is a degree of selective non-participation of individuals from opposite ends of a spectrum: on the one hand, the youngest age band (non-response to Health Survey), currently in employment, and with minor episodes of knee pain, and, on the other, the most elderly, who are more likely to have persistent or severe knee pain and other morbidity.

Yet despite the level of non-participation, our main finding is that it may have only a modest effect on the cross-sectional distribution of key variables (e.g. WOMAC scores) and prevalence odds ratios (e.g. between physical function and obesity). The subgroup of individuals who attended the research clinics and who make up the CAS(K) cohort can be used to estimate these parameters accurately in the reference population as a whole. The main effect of non-participation in the current cohort is likely to be a loss of precision in stratum-specific estimates e.g. in those aged 80 years and over. The additive effects of non-response and non-consent are therefore still important [21] but strategies such as oversampling minority groups or those anticipated to have higher levels of non-participation (e.g. [22]) may remedy this in future studies.

We used a number of strategies that have been shown to increase response to postal surveys [23]. These included the use of pre-pilot and pilot studies to make the questionnaires more interesting and user-friendly, University sponsorship, the omission of sensitive questions (e.g. income, recent life events), reminder postcard and repeat questionnaire mailing to initial non-respondents, request of an explanation for non-participation (voluntary), placement of relevant knee-specific questions at the start and general questions at the end of the questionnaire (18-month follow-up only), and postal pre-contact (18-month follow-up only). Conversely, our postal questionnaires were long (due to both the scope of data collection and font size felt necessary for this age group); we offered no financial or other personal incentives; we used standard delivery with business reply, and all respondents to the baseline Health Survey were offered the ability to opt out of further contact or medical record review. These strategies may reduce response rates. In the current study, these choices were driven by ethical and cost considerations as well as the ambitious scope of the study. A trade-off may well exist between non-participation at baseline and subsequent loss to follow-up. Attrition at 18 months amongst CAS(K) clinic attenders was very low (3%), similar to a comparable study recently conducted in the United States [24]. The inclusion of the tracing service and minimum data collection at the 18-month follow-up was valuable. Though it directly contributed only 3% to the adjusted response at 18-months (24/802), it halved the level of attrition from 6% (50/802) to 3% (26/802).

We have considered the representativeness of the CAS(K) cohort from the perspective of the sampling frame chosen; that is, adults aged 50 years and over registered with three general practices in North Staffordshire and experiencing knee pain within the previous 12 months. Very few of the target population were from ethnic minorities. We have investigated the effects of selective non-participation and attrition on cross-sectional parameters (descriptive characteristics, prevalence odds ratio). Whilst our findings provide some reassurance on the generalisability of findings from this cohort, we recognise that this cannot be assumed to apply to all subsequent associations and outcomes studied within this cohort. Bias resulting from loss to follow-up, self-selection, and missing data can occur despite the best efforts of investigators [25] and the role of this in each subsequent analysis must be considered on its own merits. In particular, the question of whether CAS(K) participants differ from non-participants in their prognosis remains unanswered. We intend to determine this at 3-year follow-up, where non-participants to the clinical assessment who consented to further contact will be followed up on the same measures as CAS(K) participants.

## Conclusion

Demographic, socioeconomic and health-related factors appeared to influence participation. Beliefs about the seriousness of the condition under investigation or the effectiveness of health care did not. In this study we found substantial non-participation but this did not introduce significant bias to cross-sectional population parameters. Findings from the CAS(K) cohort can be generalised to the target population from which they were drawn although investigating the possible biasing effects of non-participation and attrition remain an important consideration for future analyses of this cohort.

## Competing interests

The author(s) declare that they have no competing interests.

## Authors' contributions

All authors participated in the design of the study and drafting the manuscript. All authors read and approved the final manuscript.

## Pre-publication history

The pre-publication history for this paper can be accessed here:



## Figures and Tables

**Table 1:** Age, gender and practice at each selection point.

	All eligible participants	Responded to Health Survey	Reported knee pain in last 12 months	Consented to further contact	Responded to Regional Pains Survey	Attended research clinic	Responded to 18 m FUP Survey
N	8984	6108	3106	2226	1949	819	776

Practice:							
A	3449 (38)	2268 (37)	1227 (40)	804 (36)	690 (36)	302 (37)	284 (37)
B	3696 (41)	2535 (42)	1251 (40)	913 (41)	806 (41)	330 (40)	314 (40)
C	1839 (20)	1305 (21)	628 (20)	509 (23)	453 (23)	187 (23)	178 (23)

Age (years):							
50–59	3014 (34)	1866 (31)	898 (29)	730 (33)	614 (32)	236 (29)	231 (30)
60–69	2535 (28)	1872 (31)	964 (31)	734 (33)	663 (34)	312 (38)	303 (39)
70–79	2056 (23)	1555 (25)	822 (26)	551 (25)	490 (25)	222 (27)	201 (26)
80+	1369 (15)	815 (13)	422 (14)	211 (9)	182 (9)	49 (6)	41 (5)

Gender							
Female	4887 (54)	3416 (56)	1832 (59)	1259 (57)	1108 (57)	440 (54)	417 (54)
Male	4097 (46)	2692 (44)	1274 (41)	967 (43)	841 (43)	379 (46)	359 (46)

Figures represent numbers and percentages unless otherwise stated

**Table 2:** Demographic, general health, psychological and lifestyle characteristics at each selectionpoint

	Responded to Health Survey	Reported knee pain in last 12 months	Consented to further contact	Responded to Regional Pains Survey	Attended research clinic	Responded to 18 m FUP Survey
N*	6108	3106	2226	1949	819	776

Marital status:						
Married/co-habiting	3956 (66)	1985 (65)	1511 (69)	1331 (69)	599 (74)	573 (75)
Divorced/separated	431 (7)	219 (7)	175 (8)	145 (8)	45 (6)	42 (5)
Widowed	1263 (21)	705 (23)	418 (19)	363 (19)	137 (17)	123 (16)
Single	358 (6)	153 (5)	93 (4)	84 (4)	27 (3)	27 (4)

Higher education:						
Yes	704 (12)	327 (11)	278 (13)	244 (13)	117 (15)	112 (15)
No	5231 (88)	2685 (89)	1892 (87)	1657 (87)	684 (85)	650 (85)

Employment status:						
Employed	1515 (26)	668 (22)	544 (25)	460 (24)	167 (21)	160 (21)
Retired	3361 (57)	1760 (59)	1195 (55)	1075 (57)	481 (61)	449 (60)
Unable due to illness	454 (8)	299 (10)	224 (10)	182 (10)	76 (10)	76 (10)
Unemployed	61 (1)	31 (1)	26 (1)	25 (1)	9 (1)	9 (1)
Housewife	362 (6)	187 (6)	129 (6)	118 (6)	46 (6)	44 (6)
Other	133 (2)	53 (2)	41 (2)	30 (2)	15 (2)	15 (2)

Occupational class:						
Higher manag.	184 (3)	92 (3)	81 (4)	78 (4)	55 (7)	53 (7)
Higher prof.	105 (2)	39 (1)	35 (2)	32 (2)	16 (2)	16 (2)
Lower manag./prof.	652 (12)	303 (11)	267 (13)	240 (13)	122 (16)	115 (16)
Intermediate	615 (11)	302 (11)	243 (12)	218 (12)	108 (14)	104 (14)
Self-employed	319 (6)	174 (6)	144 (6)	124 (7)	52 (7)	49 (7)
Lower supervise./tech.	387 (7)	213 (8)	151 (7)	140 (8)	63 (8)	61 (8)
Semi-routine	1397 (25)	712 (25)	505 (25)	443 (25)	190 (25)	177 (24)
Routine	1887 (34)	979 (35)	628 (31)	532 (29)	167 (22)	156 (21)

Baseline SF-12: Mean (sd†)						
Physical (0–100)	41.0 (12.5)	37.0 (12.2)	37.6 (12.2)	37.7 (12.2)	37.6 (11.9)	37.6 (11.8)
Mental (0–100)	49.1 (11.1)	47.9 (11.6)	48.5 (11.5)	48.8 (11.4)	50.6 (10.9)	50.8 (11.0)

Baseline HAD Anxiety:						
None (0–7)	3647 (62)	1656 (55)	1209 (56)	1084 (57)	500 (62)	477 (63)
Possible (8–11)	1485 (25)	842 (28)	603 (28)	526 (28)	217 (27)	203 (27)
Probable (12–21)	791 (13)	508 (17)	343 (16)	284 (15)	85 (11)	81 (11)

Baseline HAD Depression:						
None (0–7)	4637 (78)	2198 (73)	1627 (75)	1456 (77)	663 (83)	633 (83)
Possible (8–11)	950 (16)	593 (20)	392 (18)	320 (17)	102 (13)	92 (12)
Probable (12–21)	349 (6)	221 (7)	139 (6)	119 (6)	37 (4)	36 (5)

"Osteoarthritis is a serious condition"						
Strongly disagree	44 (1)	19 (1)	12 (1)	12 (1)	4 (1)	4 (1)
Disagree	67 (1)	33 (1)	25 (1)	22 (1)	8 (1)	8 (1)
Neither	423 (7)	189 (6)	134 (6)	108 (6)	39 (5)	36 (5)
Agree	3326 (56)	1656 (55)	1163 (53)	1032 (54)	447 (56)	421 (55)
Strongly agree	2074 (35)	1129 (37)	847 (39)	740 (39)	305 (38)	292 (38)

"Doctors can do a lot to help people with joint pain"						
Strongly disagree	50 (1)	30 (1)	25 (1)	20 (1)	9 (1)	9 (1)
Disagree	408 (7)	251 (8)	197 (9)	172 (9)	74 (9)	71 (9)
Neither	1331 (22)	659 (22)	483 (22)	431(22)	171 (21)	163 (21)
Agree	3693 (62)	1851 (61)	1300 (59)	1142 (59)	482 (60)	456 (60)
Strongly agree	510 (9)	261 (9)	191 (9)	160 (8)	73 (9)	67 (9)

Figures represent numbers and percentages unless otherwise stated

* – individual items may not add to totals due to missing data

† – sd = standard deviation

**Table 3:** Knee pain characteristics at each selection point.

	Responded to Regional Pains Survey	Attended research clinic	Responded to 18 m FUP Survey
N*	1949	819	776

Baseline knee pain chronicity:			
Less than 7 days	180 (11)	61 (8)	58 (8)
1–4 weeks	249 (15)	106 (14)	103 (14)
1 mo to < 3 mo	252 (15)	117 (16)	111 (16)
3 mo+	1019 (60)	469 (62)	441 (62)

Baseline WOMAC: Mean (sd†)			
Pain (0–20)	6.3 (4.5)	6.4 (4.4)	6.4 (4.4)
Stiffness (0–8)	2.7 (2.0)	2.7 (1.9)	2.7 (1.9)
Function (0–68)	21.1 (15.8)	20.9 (15.2)	20.7 (15.2)

Baseline radiographic knee OA¶:			
Definite (any compartment)			
Yes		539 (68)	514 (69)
No		251 (32)	235 (31)

Definite (patello-femoral) ¶:			
Yes		507 (64)	481 (64)
No		283 (36)	268 (36)

Definite (tibia-femoral) ¶:			
Yes		350 (44)	332 (44)
No		440 (56)	417 (56)

Severe (any compartment) ¶:			
Yes		313 (40)	293 (39)
No		477 (60)	456 (61)

Baseline symptomatic knee OA‡			
Yes		260 (33)	242 (32)
No		530 (67)	507 (68)

Figures represent numbers and percentages unless otherwise stated

* – individual items may not add to totals due to missing data

† – sd = standard deviation

¶ – Baseline x-ray data is available for 790 participants at recruitment

‡ – Knee pain, aching or stiffness on most days in the last month plus definite radiographic osteoarthritis in the index knee

**Table 4:** Baseline WOMAC scores in CAS(K) clinic attenders and population norms.

	Pain (0–20)		Stiffness (0–20)		Physical Function (0–68)	
	CAS(K)	Population norm†	CAS(K)	Population norm†	CAS(K)	Population norm†

Male						
50–64 years	5.8 (4.4)	5.5 (4.3)	2.4 (2.1)	2.5 (2.0)	19.2 (15.7)	17.6 (15.4)
65–74 years	6.4 (4.2)	6.5 (4.5)	2.7 (1.8)	3.1 (2.0)	20.6 (14.7)	22.6 (15.4)
75+ years	7.5 (3.9)	7.2 (4.3)	3.0 (1.8)	3.1 (1.9)	24.9 (13.9)	25.9 (15.2)

Female						
50–64 years	5.9 (4.5)	6.1 (4.5)	2.5 (1.9)	2.7 (2.1)	18.8 (15.5)	18.8 (16.0)
65–74 years	7.0 (4.4)	7.2 (4.5)	2.9 (1.9)	3.1 (2.0)	23.0 (15.0)	24.4 (15.6)
75+ years	6.7 (4.2)	6.6 (4.5)	2.9 (2.0)	2.9 (2.0)	23.0 (14.9)	22.2 (16.4)

† From [17]

**Table 5:** Main factors associated with participation in research clinics compared with observed target population.

	Reported knee pain in last 12 months	Attended research clinic	OR (95%CI)
N*	3106	819	

Practice			
A (ref.)	1227	302	1
B	1251	330	1.10 (0.92, 1.31)
C	628	187	1.30 (1.05, 1.61)

Age (years):			
50–59 (ref.)	898	236	1
60–69	964	312	1.34 (1.10, 1.64)
70–79	822	222	1.04 (0.84, 1.29)
80+	422	49	0.37 (0.26, 0.51)

Gender:			
Female (ref.)	1832	440	1
Male	1274	379	1.34 (1.14, 1.57)

Marital status:			
Married (ref.)	1985	599	1
Divorced/separated	219	45	0.60 (0.43, 0.84)
Widowed	705	137	0.56 (0.45, 0.69)
Single	153	27	0.50 (0.32, 0.76)

Higher education:			
Yes (ref.)	327	117	1
No	2685	684	0.61 (0.48, 0.78)

Occupational class†:			
Non-manual (ref.)	736	301	1
Manual	1904	420	0.41 (0.34, 0.49)

Baseline HAD Anxiety:			
None (0–7) (ref.)	1656	500	1
Possible (8–11)	842	217	0.80 (0.67, 0.97)
Probable (12+)	508	85	0.46 (0.36, 0.60)

Baseline HAD Depression:			
None (0–7) (ref.)	2198	663	1
Possible (8–11)	593	102	0.48 (0.38, 0.61)
Probable (12+)	221	37	0.47 (0.32, 0.67)

* – individual items may not add to totals due to missing data

† – non-manual consists of higher managerial, higher professional, lower managerial/professional, and intermediate occupations; manual consists of lower supervisory/technical, semi-routine, and routine occupations. Self-employed have been excluded.

**Table 6:** Association between body mass index and SF-36 physical function in observed target population and CAS(K) clinic attenders*

	Poor physical function	Good physical function	Odds ratio (95% CI†) Unadjusted	Odds ratio (95% CI†) Adjusted‡
Reported knee pain in last 12 months (n = 3106)				

Self-reported body mass index				
Underweight/normal (<25·0 kg/m^2^)	550	321	1.00	1.00
Overweight (25·0–29·9 kg/m^2^)	750	420	1.04 (0.9, 1.3)	1.38 (1.1, 1.7)
Obese (>29·9 kg/m^2^)	520	164	1.85 (1.5, 2.3)	2.71 (2.1, 3.4)

Attended research clinic (n = 819)				

Self-reported body mass index				
Underweight/normal (<25·0 kg/m^2^)	100	83	1.00	1.00
Overweight (25·0–29·9 kg/m^2^)	216	136	1.32 (0.9, 1.9)	1.41 (1.0, 2.1)
Obese (>29·9 kg/m^2^)	164	46	2.96 (1.9, 4.6)	3.64 (2.3, 5.8)

* Both SF-36 physical function scores and self-reported body mass index are subject to missing data.

† – 95% confidence interval

‡ – Adjusted for gender and age-group (50–59 yrs, 60–69 yrs, 70–79 yrs, 80+ yrs)
